# Unorganised: Its Difficulties and a Pernicious System

**Published:** 1917-03-03

**Authors:** 


### NURSE TRAINING IN IRELAND.

### Unorganised: Its Difficulties and a Pernicious System.

THE entirely unorganised and chaotic condition of nursing in Ireland is nowhere more obvious and deplorable than in the case of the candidate probationer, whose plight is particularly unfortunate. Some hospitals charge a fee of fifty guineas for training, others twenty-five, others ten, while one or two are free. These sums, be it observed, form no guide to the status or efficiency of the institution; indeed, in many cases the less expensive are the best. Then it happens that in some cases a small salary is paid to the probationer; in others there is none, at all events for the first year, and in no case is the salary sufficient to cover necessary expenses of dress, not to mention holidays and travelling. The principle adopted by the hospitals, consciously or unconsciously, would appear to be that workers in a charitable institution should be paid a charity wage, and that the nurse-probationer is taken account of in the announcement to be found in the hall, "This institution is supported by voluntary contributions." There is a tale told of a lady Church worker who asked the village shoemaker to supply a pair of boots "cheap—as they are required in the cause of charity." The shoemaker, however, being a shrewd man, replied, "I always do my own charity, Miss!" The humble nurse-probationer who pays a substantial training fee and then toils for three or four years for nominal remuneration, may not have her name inscribed in the list of donors, but she is none the less a liberal contributor. She receives no credit for her philanthropy, nor for the menial duties which she is bound to perform. On the contrary, she is taught that she is receiving a valuable training, the fruit of which she is bound to gather in due season.

### IMPORTED MATRONS.

It is a very serious matter for a young woman in the twenties to find herself the victim of this pernicious system. Her hours of duty are long, her duties arduous and responsible, and her conditions of employment in every respect inferior to those of a domestic servant. Perhaps the worst feature of the case is that usually the last person to discover the truth is the probationer herself, who learns only when too late that Dublin hospitals as training-schools do not compare favourably with those of London, Liverpool, or Manchester. This applies, of course, especially to employment outside Ireland, but it is a fact not a little remarkable that even in Dublin it is becoming fashionable to fill vacant matronships by nurses trained in Great Britain. This is perfectly natural. It must of necessity happen that new discoveries, new methods, begin with the Metropolis and gradually extend to the provinces, and, of course, in respect of medicine and science generally Ireland is distinctly provincial.

A Retrogressive Policy.

Hitherto the hospitals have enjoyed the advantage. Aided by the probationers' fees and practically free labour, they have been run on very inexpensive lines. It is doubtful, however, if this will continue. The poor professional man who cannot afford a Dublin training for his daughters has discovered that great London institutions like the London Hospital make no charge, or at most a very small charge, and that probationers immediately begin to earn reasonable salaries, progressing each year. The result is, as London matrons will testify, that Irish girls of breeding and refinement are coming to London in much larger numbers than used to be the case, whilst a constantly increasing number of half-educated girls, drawn from a moneyed but less refined stratum of society, are attaching themselves to the Irish hospitals. There is no entrance examination, the great desideratum being ability to pay. The policy is penny wise, pound foolish, and sooner or later must react prejudicially not alone on the hospitals but on the nursing profession in this country.

The Difficulties in the Way.

There is a very real difficulty in the way. In Ireland there is a peculiar and perhaps a justifiable prejudice against girls going too far from home, more especially to an appointment where remuneration in the best of circumstances is low. This prejudice is buttressed by the fact that no matron will accept a probationer without having interviewed her and taken her measure as a probable success or failure. No postal communication, even with photographs, can be regarded as the equivalent of a conversation face to face. Hence the candidate probationer finds it necessary, if she would enter a hospital in Great Britain, to go thither and take her chance of finding a willing matron in a hospital where there are vacancies. The result is that after much cogitation "the devil you know" is preferred, the big fee is paid, and the girl remains in Ireland.

A Remedy.

It would appear as if the establishment in Ireland of the College of Nursing might afford a suitable opportunity for remedying this primitive condition of affairs. The Irish Nurses' Association do not appear to have ever made any effort in the direction of reform, nor, indeed, to have become properly awake until the College came into being. Is it not possible for the College in London to provide an arrangement whereby the Irish Board, when established, should interview nurse candidate probationers and certify them as good or otherwise? They might be graded as first, second, or third class, according to their physique, their educational attainments and accomplishments. This would have a marked effect on Ireland. It would, in the first place, confer an immense boon on Irish girls; in the second, it would cause the Irish hospitals to amend their methods, and, in addition, it would demonstrate to the nursing profession in Ireland the intrinsic value of the College. It is too often forgotten that in order to raise the status of any profession it is necessary to begin with the entrant.

IERNE.

